# EARLY ILEOCECAL RESECTION IN CROHN’S DISEASE: A SYSTEMATIC REVIEW

**DOI:** 10.1590/S0004-2803.24612025-013

**Published:** 2026-01-09

**Authors:** Alexandra Damasio TODESCATTO, Abel Botelho QUARESMA, Paulo Gustavo KOTZE

**Affiliations:** 1 Pontifícia Universidade Católica do Paraná (PUCPR), Curitiba, PR, Brasil.; 2 Universidade do Oeste de Santa Catarina, UNOESC, ambulatório de DII, Joaçaba, SC, Brasil.; 3 Pontifícia Universidade Católica do Paraná, Programa de Pós-Graduação em Ciências da Saúde, Curitiba, PR, Brasil.

**Keywords:** Crohn’s disease, surgery, inflammatory bowel disease, early intestinal resection, drug treatment, Doença de Crohn, cirurgia, doença inflamatória intestinal, ressecção intestinal precoce, tratamento medicamentoso

## Abstract

**Background:**

The approval of new therapies has led to an increasing trend toward the use of various combinations of medications in Crohn’s disease (CD) management. However, patients with ileocecal CD may still require surgery in up to 80% of cases.

**Objectives:**

To analyze the literature and synthesize the data qualitatively to evaluate the outcomes of early surgery compared to drug therapy in patients with ileocecal CD, focusing on both adult and pediatric populations.

**Methods:**

Studies were selected through an electronic search of the PUBMED database, following the PRISMA (Preferred Reporting Items for Systematic Reviews and Meta-Analyses) guidelines. The MINORS (Methodological Index for Non-Randomized Studies) criteria were used to assess the methodological quality of non-randomized studies.

**Results:**

A total of 665 articles were initially identified through the search strategy, and three additional relevant articles were added manually, leading to 22 studies eligible for qualitative evaluation. The evaluation of results was organized based on primary outcomes. Three studies assessed postoperative morbidity, with two showing higher morbidity in patients who underwent surgery for CD complications compared to those operated on for a purely inflammatory phenotype. Twelve observational studies evaluated CD recurrence, with ten showing evidence of higher surgical recurrence in patients who underwent surgery at a later stage. These studies demonstrated worse long-term clinical control of CD in this group, with a higher need for corticosteroids and advanced therapies. Only two pediatric studies met the inclusion criteria, limiting a more comprehensive analysis of this population.

**Conclusion:**

Early surgery in adult patients is a solid therapeutic option in the treatment of uncomplicated isolated ileocecal CD.

## HIGHLIGHTS

•Early ileocecal resection is a safe and effective therapeutic option for adult patients with uncomplicated Crohn’s disease.

•Patients undergoing early surgery present lower morbidity compared with those treated surgically for complicated disease phenotypes.

•Surgery performed before complications arise results in technically less demanding procedures and fewer reoperations.

•Long-term follow-up demonstrates superior disease control and sustained remission in patients undergoing early resection.

•Early ileocecal resection in pediatric Crohn’s disease may contribute to sustained remission and improved growth outcomes, yet the paucity of evidence highlights the necessity for well-designed prospective trials in this population.

## RESUMO

**Contexto:**

A aprovação das novas terapias resultou numa tendência à utilização de diferentes combinações de medicamentos, entretanto, pacientes com doença de Crohn (DC) ileocecal podem precisar de cirurgia em quase 80% dos casos.

**Objetivo:**

Analisar a literatura e sintetizar qualitativamente os dados a fim de se avaliar os desfechos da cirurgia precoce em comparação à terapia medicamentosa em pacientes com DC ileocecal na população adulta e pediátrica.

**Métodos:**

Os estudos foram selecionados através de busca eletrônica na base de dados do PUBMED de acordo com o guideline PRISMA (Preferred Items of Reports for Systematic Reviews and Meta-Analysis). Para avaliação da qualidade metodológica dos estudos não randomizados, foram utilizados os critérios MINORS (Methodological index for non-randomized studies).

**Resultados:**

665 artigos foram inicialmente encontrados pela estratégia de busca e outros três artigos relevantes foram adicionados manualmente, sendo 22 estudos elegíveis para a avaliação qualitativa. A avaliação dos resultados foi alocada conforme os desfechos primários. Três artigos avaliaram morbidade pós-operatória, as quais foram maiores em dois desses estudos nos pacientes operados por complicações da DC, em comparação a pacientes operados por doença estritamente inflamatória. Doze estudos avaliaram a recorrência da DC, todos observacionais, sendo que em dez há evidência de maior recorrência cirúrgica em pacientes operados mais tardiamente. Os estudos demonstraram pior controle clínico da DC a longo prazo nesse grupo de pacientes, com maior necessidade de corticoide e terapias avançadas. Apenas dois estudos pediátricos atenderam aos critérios de inclusão, não permitindo análise mais complexa nessa população.

**Conclusão:**

A ressecção ileocecal em pacientes adultos é uma sólida opção terapêutica no tratamento da DC ileocólica não complicada.

## INTRODUCTION

Crohn’s disease (CD) is a chronic, idiopathic inflammatory bowel disease (IBD) that negatively impacts patients’ quality of life and increases morbidity over time^1^. It can affect any part of the digestive tract, but in most patients, it is located in the terminal ileum and may extend to the colon^2^.

Current therapeutic strategies for CD primarily focus on modulating the hyperactive immune response using immunosuppressive drugs^3^. Despite the introduction of new therapies, up to 50% of patients treated with these agents show no significant improvement in clinical symptoms, and many experience mild to severe side effects^4^ ^-^ ^5^. More advanced therapies, such as vedolizumab and ustekinumab, have demonstrated lower rates of adverse events and good therapeutic efficacy^6^ ^-^ ^8^. However, the discontinuation rates remain high, either due to lack of initial response or loss of response over time. Furthermore, patients who do not respond to anti-TNF agents, commonly used as the first-line treatment, tend to have lower success rates with second-line biologics^8^ ^-^ ^10^.

Ileocecal resection (ICR) has traditionally been reserved for patients with complications of CD (e.g., stenosing or penetrating disease) or for those who are refractory to medical therapy. The approval of advanced therapies has led to a growing preference for medical treatment combinations, often relegating surgery to a last-resort option^11^ ^-^ ^12^. However, reports suggest that nearly 80% of patients with ileocecal CD eventually require surgery at some point during their disease course^13^.

While patients with complicated disease clearly benefit from surgical treatment, the management of uncomplicated CD (characterized solely by inflammation - luminal phenotype) remains a topic of debate^14^ ^,^ ^15^. Early surgical intervention in predominantly inflammatory CD of the terminal ileum or ileocecal region aims to alter the natural disease course by inducing remission, improving quality of life, and reducing the risk of complications^2^ ^,^ ^16^ ^,^ ^37^. Long-term outcomes suggest that surgery leads to rapid remission, fewer complications, and is more cost-effective compared to anti-TNF therapy, prompting a reevaluation of current treatment protocols^17^.

Recent evidence supports surgery as a potential first-line treatment option. For example, a Danish prospective cohort study compared the outcomes of patients treated with ICR versus those on anti-TNF therapy within the first year of diagnosis. The study found that patients on biologics had lower rates of corticosteroid use and fewer CD-related surgeries. Additionally, in the surgical group, approximately 50% of patients did not require drug therapy at the five-year follow-up^17^.

Given the ongoing debate about the optimal timing for surgical intervention in uncomplicated luminal CD, this review aims to conduct a qualitative systematic analysis of surgical outcomes in early resection for ileocecal CD. The goal is to inform clinical decision-making and treatment strategies based on the most current scientific evidence, thereby improving patient care.

## METHODS

This qualitative systematic review (QSR) was conducted in accordance with the Preferred Reporting Items for Systematic Reviews and Meta-Analyses (PRISMA) guidelines^18^. A complete (unregistered) protocol for the QSR was designed to achieve the objectives.

### Search Strategy

The included studies were identified through an electronic search in the Medline database via PubMed [https://www.ncbi.nlm.nih.gov/pubmed/]. A comprehensive bibliographic search was performed on October 31, 2023, using Health Sciences Descriptors (DeCS), which were developed from the Medical Subject Headings (MeSH) of the US National Library of Medicine (NLM). The following search sequence was used: (Early [Title] OR luminal [Title] AND surgery OR ileocecal resection AND Crohn [Title]).

### Eligibility criteria

Randomized clinical trials, case-control studies, and retrospective and prospective cohort studies evaluating early surgery for luminal CD in adult or pediatric patients were included. Comparisons with drug therapy or later surgery were also accepted. Only full-text studies published in English were eligible. Early surgery was defined as surgery performed within one year of diagnosis or in the presence of strictly luminal disease (purely inflammatory phenotype), irrespective of the time since diagnosis.

### Outcomes

The primary outcomes evaluated were postoperative morbidity and clinical, endoscopic, or surgical recurrence.

### Literature review and data extraction

Two authors (ADT and ABQ) independently reviewed the search results and selected potentially relevant studies based on titles. The results were compared, and any discrepancies between the two were resolved by a third author (PGK). After excluding articles that did not meet the inclusion criteria based on their titles, the abstracts were assessed. This was followed by a full-text evaluation to determine which studies should be included. Relevant studies not identified through the search strategy were manually added.

### Assessment of methodological quality and risk of bias

The methodological quality of the studies was assessed using the Methodological Index for Non-Randomized Studies (MINORS)^19^. Items were scored as 0 (not reported), 1 (reported but inappropriately), or 2 (reported appropriately). The ideal score is 16 for non-comparative studies and 24 for comparative studies.

## RESULTS

**FIGURE 1:**
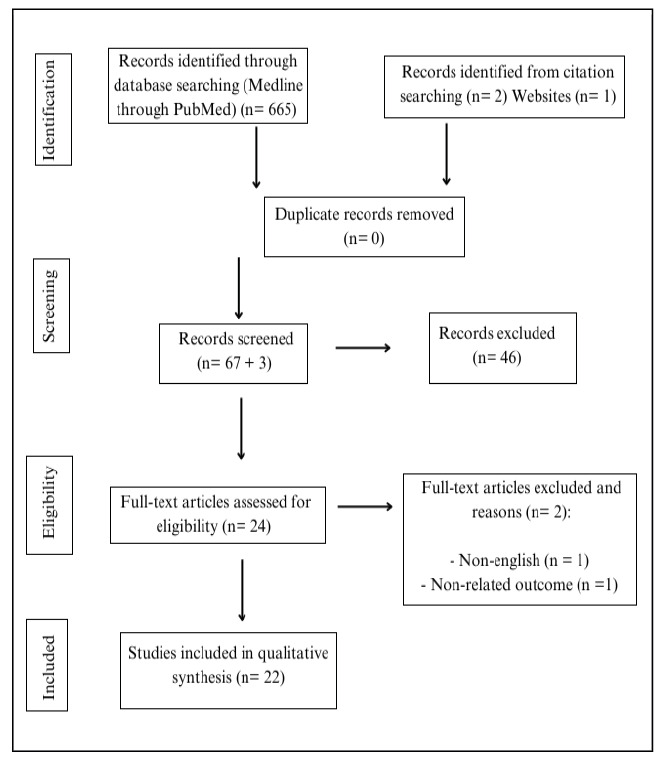
Systematic review PRISMA flow diagram^18^.

A total of 665 articles were initially identified using the PubMed database search strategy. An additional three relevant articles were manually added, resulting in 668 articles considered for evaluation. After applying the inclusion and exclusion criteria, 24 articles were selected for full-text reading. Of these, two were excluded, leaving 22 articles for qualitative analysis. The flowchart detailing the inclusion process is illustrated in Figure 1. The evaluation of the MINORS criteria is presented in Table 1.

**TABLE 1:** The methodological quality of the studies was assessed using the methodological index for non-randomized studies (MINORS)^19^. The items are scored 0 (not reported), 1 (reported, but inadequate), or 2 (reported and adequate). The global ideal score was 16 for non-comparative studies and 24 for comparative studies.

MINORS^19^	Avellaneda et al. 2022^20^	Avellaneda et al. 2023/1^21^	Overstraeten et al. 2017^22^	Beelen et al. 2023^23^	Avellaneda et al. 2023/2^24^	An V et al. 2016^25^	Golovics et al. 2013^26^	Aratari et al. 2007^27^	Sarikaya et al. 2022^28^	Kelm et al. 2021^29^	Agrawal et al. 2023^17^	Dias et al. 2018^30^	Latella et al. 2009^31^	Lee et al. 2018^32^	Magro et al. 2017^33^	Saeed et al. 2017^34^	Hojsak et al. 2012^35^	Cook et al. 2007^36^
**1. A clearly stated aim**	2	2	2	2	2	2	2	2	2	2	2	2	2	2	2	2	2	2
**2. Inclusion of consecutive patients**	2	2	2	2	2	2	1	2	2	2	2	2	2	2	2	1	2	2
**3. Prospective collection of data**	0	0	0	1	2	2	2	1	2	1	1	1	1	1	1	2	1	1
**4. Endpoints appropriate to the aim the study**	2	2	2	2	2	2	2	2	2	2	2	2	2	2	2	2	2	1
**5. Unbiased assessment of the study endpoint**	2	0	0	0	0	0	0	0	0	0	0	0	0	0	0	0	0	0
**6. Follow-up period appropriate to the aim the study**	2	2	2	2	2	2	2	2	2	2	2	2	2	2	2	2	2	2
**7. Loss to follow-up less than 5%**	0	0	0	0	2	1	0	0	0	0	0	0	0	1	2	2	2	1
**8. Prospective calculation of the study size**	0	0	0	0	2	0	0	0	0	0	0	0	0	0	0	0	0	0
**Additional criteria in the case of comparative study**																		
**9. An adequate control group**	2	2	-	-	-	2	-	1	1	1	2	-	1	2	1	-	-	-
**10. Contemporary groups**	2	2	-	-	-	2	-	2	2	2	2	-	2	2	2	-	-	-
**11. Baseline equivalence of groups**	2	2	-	-	-	1	-	2	1	2	2	-	2	2	2	-	-	-
**12. Adequate statistical analyses**	2	2	-	-	-	1	-	2	2	1	2	-	2	2	2	-	-	-
**TOTAL**	16	16	8	9	14	17	9	16	16	15	17	9	16	18	18	11	11	9

### Postoperative complications

The studies that assessed postoperative complications as the primary outcome are summarized in Table 2. All studies were observational and retrospective, with the number of participants ranging from 273 to 538 patients.

**TABLE 2:** Main outcome Analyzed: postoperative mobility.

Author	Journal	Groups	N	Operative time (mean - minuts)	Major post-operative complications	Anastomotic leak	Adicional procedures	Length of stay (mean - days)
**Avellaneda** **et al. 2022** ^20^	Journal of Clinical Medicine	ECD	60 (17,8)	90.53	40%	1.67%	3.33%	6.48
		CCD	277 (82,2)	164.25	51.09%	6.67%	13.36%	8.33
**Avellaneda** **et al. 2023/1** ^21^	Digestive and Liver Disease	ECD	85 (31)	115	9.41%	3.53%	1.18%	5.45
		CCD	188 (69)	126	14.89%	6.82%	19.15%	7.40
**Overstraeten et al. 2017** ^22^	British Journal of Surgery		538	127	4.8%	3%	175 (32.5%)	7

ECD = early Crohn’s disease. CCD = complicated Crohn’s disease.

Two of the three articles were comparative studies^20^ ^-^ ^21^. In both studies, the rate of postoperative complications, as classified by the Clavien-Dindo classification (CCD >IIIa^20^ and CCD >II^21^), was numerically higher in patients undergoing surgery for complications of CD, compared to those undergoing early surgery for luminal disease. The average operative time, conversion rate to open surgery, and need for additional procedures were significantly higher in patients with stenosis and perforation. Additionally, patients in the complicated disease group had a higher incidence of emergency surgery and lower rates of primary anastomosis. Although the average length of stay was numerically higher in the complicated CD group, it was not statistically significant: 6.48 vs 8.33 days (*P*=0.208)^20^ and 5.45 vs 7.40 days (*P*=0.093)^21^.

The cohort study by Overstraeten et al.^22^ included patients undergoing primary surgery, with 42.9% of the patients presenting sepsis at the time of surgery. The conversion rate was 12.3%, similar to the rates in the other two studies (10.4% and 13.19%). In terms of postoperative complications, 4.8% of patients experienced Clavien-Dindo morbidity ≥IIIa. Approximately one-third of patients required additional procedures. Surgical recurrence occurred in 6.5% and 19.1% of patients at 5 and 10 years, respectively, with smoking and positive microscopic surgical margins being significant risk factors for recurrence.

### Postoperative Recurrence

Twelve studies evaluated recurrence as the primary outcome, which could be clinical, endoscopic/radiological, or surgical recurrence. The main data from these studies are summarized in Table 3. All the studies were observational, with eight of them being comparative. The number of participants ranged from 103 to 2,483, and the studies included patients treated between 1977 and 2019. Only one study^24^ exclusively evaluated patients with non-penetrating, non-stenosing disease (B1).

**TABLE 3:** Main outcome Analyzed: recurrence.

Author/ year	Inclusion period	Groups	N	Endoscopic or radiologic recurrence	Switch or drug optimization	Reoperation	Montreal Classification
**Belen et al.** **2023** ^23^	2000-2019		822	0 m: 34.6%	20.2%	19.8%	L1 and L3 B1-B3
				4m: 48.2%	28.8%	29.8%	
				12m: 39%	28.3%	34%	
**Avellaneda et al. 2023/2** ^24^			211	24m: 44%		3%	B1
				48m: 48%		4%	L1 and L3
				72m: 55%		7%	
				96m: 62%		10%	
				120m: 67%		10%	
**Agrawal et al. 2023** ^17^	2003-2018	ICR	581	-	16.8% started antiTNF	1.8%	L1 and L3 B1-B3
		anti-TNF	698	-	40.8% switch	17.7% first surgery	
**Sarikaya et al. 2022** ^28^	1997-2015	29d	493	-	-	28.7%	L1-L3 B1-B3
		30-180d	472	-	-	30.5%	
		>180d	1518	-	-	34.2%	
**Kelm et al.** **2021** ^29^	2006-2017	ES	29	-	3.4%	3.4	L1-L4 B1-B3
		IMT	74	-	27%	1.4%	
**Dias et al.** **2018** ^30^	-		498	-	-	192(39%)	B1-B3 L1-L4
**Lee et al.** **2018** ^32^	1982-2008	ES	120		Lower use of biological	No difference between the groups	B1-B3 L1-L3
		LS	123	-	-		
**Magro et al. 2017** ^33^	-	ES	244	-	-	38%	L1-L4 B1-B3
		IMT	510	-	-	21% first surgery 12% reoperation	
**An V et al.** **2016** ^25^	1995-2014	ES	42	-	-	14,2%	B1-B3 L1 and L3
		IMT	115	-	-	53.9% first surgery 24.2% reoperation	
**Golovics et al. 2013** ^26^	1977-2018		506	-		14.4% ES reoperation 5y 7.5%,	L1-L4 B1-B3
						10y 16.5%	
						Late surgery:	
						2y 12,1%, 5y 33.2%,	
						10y 53.6%	
						Reoperation: 12.9%	
						5y e 36.3 10y	
**Latella et al. 2008** ^31^	1980-2005	Surgical diagnosis	115	-		5y 14% 10y 30%	B1-B3 L1-L4
		Clinical diagnosis	375	-	-	5y 30% 10y 44%	
**Aratari et al. 2007** ^27^	-	ES	83	40.1% clinical recurrence	-	16.4%	B1-B3 L1 and L3
		LS	124		-		

ES: early surgery. LS: late surgery. IMT: inicial medical treatment

The cohort study by Agrawal et al.^17^, which included patients from 2003 to 2018, is the only study that exclusively compared early surgery to anti-TNF drug therapy. Patients who underwent early ileocecal resection had lower rates of corticosteroid use and CD-related surgery. About half of the patients in the surgical group did not require drug therapy at the end of the 5-year follow-up, indicating adequate clinical and endoscopic control of the disease.

In the other studies, patients with uncomplicated disease were included alongside those with stenosing and penetrating disease (B2 and B3, respectively), with locations not restricted to the ileum and ileocecal region. Drug treatments included aminosalicylic acid (5-ASA) derivatives, corticosteroids, immunomodulators, and biologics. Surgery was performed using conventional or minimally invasive techniques, either for acute CD complications or on an elective basis. As a result, there was considerable heterogeneity among the participants in this subgroup.

Reoperation (and the need for surgery in the control groups) was the only outcome assessed in all the studies. The study by Lee et al.^32^ was the only one that showed no difference between the interventions. Kelm et al*.* ^29^ reported a numerically higher reoperation rate in the “early surgery” group, but this was not statistically significant. In patients who underwent later surgeries, the reoperation rate tended to be higher compared to those who had earlier surgery^25^ ^,^ ^26^ ^,^ ^28^ ^,^ ^31^.

Beelen et al.^23^ also showed that patients operated on within 30 days of diagnosis had a 14% lower reoperation rate compared to those who underwent surgery 12 months after diagnosis. However, there was no difference in postoperative prognosis between the groups. In this study, approximately 50% of patients underwent laparoscopic surgery. Lower rates of endoscopic recurrence and medication changes were observed in the early surgery group. Prophylactic treatment in the postoperative period was associated with lower recurrence rates, in line with other studies^24^ ^,^ ^30^ ^,^ ^33^.

Avellaneda et al.^24^ evaluated ICR in patients with an inflammatory phenotype, comparing outcomes with patients who had complications from Crohn’s Disease (CD). Endoscopic recurrence at 12 months post-surgery was 49%, and clinical recurrence at 24 months was 17%. Prophylactic treatment was considered a protective factor. Fewer than 2% of patients did not receive a primary anastomosis, and 89% underwent laparoscopic surgery. The conversion rate to open surgery was 1.6%, and 5.2% of patients had postoperative complications greater than II (Clavien-Dindo), demonstrating satisfactory intra - and post-operative outcomes for minimally invasive surgery for ICR in luminal disease.

Aratari et al.^27^ also assessed clinical recurrence. The probability of clinical recurrence at 10 years was lower in the early surgery group, but no difference in surgical recurrence was observed. Kelm et al.^29^ did not find differences in surgical recurrence, but there was a statistically significant advantage for early surgery compared to stepwise drug therapy. At a 2-year follow-up, 77% of patients in the clinical treatment group required immunosuppressive therapy, compared to 37.9% in the early surgery group. Additionally, the early surgery group experienced a longer interval before the need to initiate therapy after surgery.

An et al.^25^ included the same proportion of patients with stenosing and penetrating phenotypes in both groups. The need for reoperation within 5 years of the first surgery was significantly higher in the “initial medical therapy” group. Furthermore, the early surgery group had lower readmission rates compared to those who underwent later surgery following initial medical therapy. Regarding medication use, there was no difference in the use of immunomodulators and corticosteroids, but the proportion of biologic use was significantly higher in the initial drug therapy group. In line with other studies^26^ ^,^ ^31^, the estimated proportion of surgeries within 10 years was also higher in the later surgery group.

Patients who underwent surgery later demonstrated greater dependence on and refractoriness to corticosteroids, with statistically significant results, as identified in the cohort by Golovics et al*.* ^26^ However, there was no difference in reoperations at 10 years among patients with exclusive terminal ileum disease who had early surgery. The lower need for biologics in the early surgery group was also identified in the study by Lee et al.^32^ The cumulative probability of biologic use over 100 months was approximately 70% vs 80% in the later surgery group. No differences were found in reoperation rates or the use of immunomodulators, but postoperative outcomes were better in the early surgery group.

In contrast, the study by Latella et al.^31^ found no difference in the use of biologics. This study stratified patients by whether they had a diagnosis of CD at the time of surgery or a clinical diagnosis. However, the first group was associated with a reduced likelihood of further surgery after the initial surgery, as well as less use of salicylates, steroids, and immunosuppressive drugs.

Another outcome assessed was the presence of disabling disease, defined by at least one of the following criteria: one or more abdominal surgeries or two hospital admissions during follow-up; dependence on or refractoriness to corticosteroids; need to switch from the first immunosuppressive or anti-TNF drug; and new clinical events after the initial episode (stenosis, perianal disease, or penetrating disease)^33^ ^,3^. Magro et al.^33^ found no significant difference between the early surgery group and the immunosuppressant group in terms of disabling disease, though the early surgery group had a higher rate of surgeries per patient. Another study also evaluated this outcome, finding that it was more closely associated with late surgery^30^.

### Other outcomes

Saeed et al.^34^ evaluated biochemical parameters in a small group of patients who underwent surgery within one year of being diagnosed with ileocecal CD. Over a mean follow-up of 13.5 months, data were collected preoperatively and postoperatively (3 - 4 weeks and at the end of follow-up). Early postoperative complications occurred in 14% of patients, but none required reoperation, and there were no late complications. There was an 85.5% and 64.3% improvement in inflammatory biomarkers (C-reactive protein [CRP] and erythrocyte sedimentation rate [ESR], respectively) compared to preoperative levels. Hemoglobin levels increased by 29.3% during the same period, with statistical significance.

### Randomized clinical trials

Two randomized clinical trials were identified, along with two follow-up studies from the LIR!C trial^15^. Thus, four articles were included for evaluation using this methodology. Gerdin et al.^38^ randomized patients with ileocecal CD to compare intestinal resection via laparotomy against corticosteroid and thiopurine treatments. The study was terminated early due to a lack of patients and because it coincided with a period of significant changes in both surgical and drug treatments. Laparoscopic surgery was found to be superior to laparotomy, and drug treatment with immunobiologics became the gold standard. At the 5-year follow-up, no clinical differences between groups were noted regarding the Crohn’s Disease Activity Index (CDAI), but the operated group showed a statistically significant improvement in social function one year post-surgery.

In 2017, the first clinical trial was published, randomizing patients to early ICR^15^ or drug treatment with IFX in patients with luminal CD. The primary outcome was quality of life, assessed via the Inflammatory Bowel Disease Questionnaire (IBDQ), while the secondary outcome was overall quality of life, measured by the Short Form-36 (SF-36) at 12 months. The IBDQ score was numerically higher, and the SF-36 score was significantly higher in the surgery group. The conversion rate to laparotomy was 7%, and 6.67% of patients had postoperative morbidity (Clavien-Dindo grade IIIa or higher). Almost 80% of patients who underwent surgery and had a colonoscopy within 12 months of surgery were in endoscopic remission. Fewer than 20% of patients in the anti-TNF group required ICR during follow-up. In 2018, the LIR!C^39^ cost-effectiveness analysis found that surgery was statistically more cost-effective compared to IFX treatment. In a 2020 long-term analysis^16^ with an average follow-up of 63.5 months, 48% of patients in the IFX group required surgery, 35% changed medication due to intolerance, and 65% required optimization due to disease activity. In the surgery group, 42% were not taking any additional medication for CD, and about half were taking immunomodulators prophylactically. None of the operated patients required additional intestinal resection.

### Studies in pediatric patients

Only two studies in the pediatric population were included for analysis (Table 4) using the MINORS quality criteria. Neither study was comparative, and both were observational. The number of patients was 68 and 37, with an average age of 12.5 and 13.7 years. The average time from diagnosis to surgery was 1.75 and 2.2 years. The multicenter study by Hojsak et al.^35^ evaluated elective ICR in pediatric patients between 2000 and 2011. The study included 68 patients, with a mean age of 13.7 at diagnosis. More than 50% of patients underwent surgery within a year of diagnosis. Around 20% showed clinical activity one year postoperatively, and over 50% remained in clinical remission at a mean follow-up of 30 months. The study demonstrated the potential for early surgery in pediatric patients with localized ileocecal CD as a therapeutic option for inducing and maintaining remission, as well as its positive impact on growth and age-adjusted body mass index (BMI) (Z score) in patients who underwent surgery before the age of 16.

**TABLE 4:** Studies in pediatric patients.

Author	Journal	N	Year	Age at diagnosis	Age at surgery	Time between diagnosis and surgery	Follow-up	Remission/ recurrence
Cook et al. 2007^36^	Journal of Crohn’s and Colitis	68	1995- 2005	12.5y	15y	1.75y	3.8y	Surgical recurrence 12.5% (12 mo)
Hojsak et al. 2012^35^	Journal of Pediatric Surgery	37	2000- 2011	13.7y	15.2y	2.2y	30mo	Remission 79.4% at 1y and 55.9% during follow-up

Cook et al.^36^ included patients who underwent elective surgery and those who had emergency surgery (3/32). Of the patients who completed follow-up, 28% required another surgery via laparotomy, with over 50% of these cases being for occlusions unrelated to CD (fibrotic stenosis of the anastomosis or occlusion caused by adhesions). There was no significant difference in the Z score for height and weight, likely due to the small sample size, which made it challenging to perform a more in-depth analysis.

### Prospective studies comparing surgery and medication:

Two prospective studies compared ileocecal resection (ICR) to anti-TNF therapy. The LIR!C study^15^ evaluated patients who underwent laparoscopic ICR versus those who received IFX as primary therapy. The primary outcome, quality of life, was higher in the surgery group. In the long-term follow-up of the LIR!C study^16^, with a mean of 63.5 months, 26% of patients who had early surgery started anti-TNF therapy, but none required additional resection. Meanwhile, 48% of patients who initially received anti-TNF therapy eventually required surgery.Agrawal et al. ^17^ analyzed the rates of a composite outcome, which included hospitalization, surgery, corticosteroid use, and the development of perianal CD over a 5-year follow-up. Patients either underwent surgery or used anti-TNF as primary treatment in the first year of diagnosis. ICR patients had a 33% lower risk of developing these events compared to those on anti-TNF therapy. In this study, around 75% of patients underwent conventional surgery (laparotomy). During the 5-year follow-up, 17.7% of those who initially received drug therapy required ICR, and 40.5% required a biologic change. Of those who underwent ICR, fewer than 2% required additional intestinal resection, and 49.7% were not on any medication by the end of follow-up. Complicated disease incidence was higher in the ICR group (21.2% vs 1.7% in the anti-TNF group).

## DISCUSSION

This systematic review included 22 studies for qualitative analysis, categorized by primary outcomes. Twelve studies evaluated recurrence, two focused on surgical outcomes in pediatric populations, two were randomized clinical trials, and three articles examined postoperative complications. The most commonly assessed primary outcome was surgical recurrence. In 10 of the 12 articles on this topic, patients who underwent early surgery had a lower need for reoperation. For postoperative complications, patients who had surgery later, with more complex disease phenotypes (e.g., stenoses and fistulas), experienced higher complication rates, longer operative times, and higher conversion rates. Despite the limited studies on pediatric populations, one study showed that early ICR had a positive impact on growth.

Prospective studies indicate that early surgery improves long-term quality of life, reduces clinical and surgical recurrence, and is more cost-effective compared to drug treatment with IFX^15^ ^-^ ^16^ ^,^ ^39^. IFX treatment in the LIR!C study appeared to delay surgery in about half of the patients. Among patients who underwent early surgery, whether by laparotomy or laparoscopy, fewer required further surgery (surgical recurrence) and corticosteroid therapy, suggesting better disease control^26^ ^,^ ^29^. A smaller proportion of patients who underwent surgery needed medication in the short to medium term compared to those who received initial drug treatment and later required surgery^24^. In patients on drug treatment, there was a greater need for therapy escalation, either through optimization of current biologics or switching to new ones^16^ ^-^ ^17^. As demonstrated by Avellaneda et al.^24^, laparoscopic ICR, when performed at experienced centers, is a safe and effective therapy that induces prolonged remission in CD, as evidenced by low conversion rates to open surgery (1.6%) and postoperative complication rates of less than 20% over 2 years.

From a surgical perspective, patients operated on early for uncomplicated disease typically have anatomically defined abdomens, resulting in technically less challenging surgeries. Studies show that these patients have shorter surgery times, lower conversion rates, and fewer postoperative complications. Additionally, patients with uncomplicated disease who undergo early surgery have a lower need for additional procedures, such as image-guided interventions or reoperations. This translates to shorter hospital stays and reduced morbidity both short- and long-term^20^ ^,^ ^24^. These advantages suggest that early surgery should be considered for patients with ileocolic CD when determining whether to pursue advanced medical therapies or surgery.

Avellaneda et al.^21^ also noted higher rates of reoperation and readmission among patients who underwent surgery for CD complications (e.g., stenosis and fistulas). These patients were typically in a more debilitated condition preoperatively, often with anemia, low preoperative albumin (requiring nutritional optimization), and previous exposure to two or more biologics. Emergency surgeries were also more common in this group.

It is well-known that histological recurrence can occur early postoperatively, and endoscopic recurrence may be seen in over 50% of patients within the first year^40^ ^-^ ^41^. However, the number of patients requiring repeat surgery for CD activity or complications is lower when surgery is performed earlier in the disease course^16^. Over the long term, patients who undergo surgery later tend to require more reoperations due to refractory disease activity and complications^17^ ^,^ ^25^ ^-^ ^26^ ^,^ ^31^. In this group, there is also a higher use of biologics, corticosteroids, and more frequent hospital readmissions, indicating more challenging disease control^16^ ^,^ ^26^ ^,^ ^29^ ^,^ ^32^. Smoking remains an important risk factor for recurrence, and prophylactic treatment is a protective factor against further surgeries^22^ ^,^ ^24^.

A current challenge in clinical practice is the lack of validated biomarkers and predictors capable of predicting response to medical therapy. If patients with a lower likelihood of responding to optimized medical treatment could be accurately identified and directed toward early surgical intervention, this could constitute a more reliable algorithm to prevent both undertreatment with medication and overtreatment with surgery. In the absence of a biomarker-driven strategy, the most appropriate current approach to managing luminal ileal CD involves individualized, multidisciplinary discussions, including IBD-specialized gastroenterologists, surgeons, and the patients themselves, in order to identify factors suggesting that the risks of delaying surgery may outweigh the potential benefits of continued advanced therapy. Nonetheless, it is essential that surgical options be discussed with patients at the same level as advanced medical therapies in all adult patients presenting with uncomplicated ileocaecal CD.

While few studies focus on pediatric populations undergoing early CD surgery, this approach remains stigmatized, often viewed as mutilating and reserved for when all clinical measures have failed. Children tend to undergo surgery later, typically after significant intestinal damage has already occurred (either through stenosing or penetrating disease), due to the long time between diagnosis and surgery^35^ ^-^ ^36^. Few data exist, and no randomized clinical trials have focused strictly on luminal disease in children. However, the multicenter study by Hojsak et al.^35^ suggests that children who undergo surgery early, with rapid surgical remission induction, fare better in terms of disease recurrence and age-adjusted growth. Early surgical intervention can result in prolonged remission and promote growth recovery, indicating that early ICR may play a critical role in pediatric and adolescent CD, though further research is needed.

Due to multiple factors, there remains a significant gap in the evidence base regarding surgical management of CD in the pediatric population. As of the time of this study, no other systematic review had included studies specifically addressing pediatric cohorts, underscoring the critical need for the generation and inclusion of evidence within this specific patient population. Moreover, only two studies-characterized by methodological heterogeneity-met the inclusion criteria for this review, which precluded the development of a standalone analysis with sufficient depth and methodological rigor. The synthesis of currently available data reinforces the need for further high-quality studies to clarify the role and outcomes of surgical interventions in pediatric CD.

This review has limitations inherent to a qualitative systematic review, including a lack of randomized clinical trials and prospective studies. The data come mainly from retrospective and observational studies, increasing the risk of bias. Furthermore, there is significant methodological heterogeneity, particularly in patient characteristics. Most studies involved patients with complicated disease, even among those with shorter disease durations, limiting evaluation of the benefits of early surgical intervention for strictly luminal disease. Despite these limitations, the findings suggest that surgery for ileocecal CD could be considered earlier as a first-line treatment, rather than only after exhausting drug alternatives or when patients are in poor clinical condition. Surgery under these circumstances is associated with worse intraoperative outcomes, increased postoperative morbidity, poorer long-term disease control, and higher healthcare costs related to drug use and hospital care.

## CONCLUSION

The findings of this review support the positive role of early ileocecal resection, performed minimally invasively, as a first-line therapeutic option for adult patients with uncomplicated CD confined to the ileocolic region. This approach should be considered during multidisciplinary discussions. In such cases, surgery has proven to be safe and is associated with favorable long-term outcomes. The morbidity of early ICR in patients with luminal inflammatory phenotypes is lower compared to those undergoing surgery for disease complications. Further prospective studies, comparing early surgery with other types of advanced therapies different than anti-TNF are encouraged to check possible differences between the two approaches in the long term. In the pediatric population, further research is also necessary to draw more definitive conclusions regarding the benefits of early surgery.

## Data Availability

Data-available-upon-request
